# Ultrasound exploration of distal posterior interosseous nerve post-surgical neuromas: a report of two cases

**DOI:** 10.1007/s00256-025-05024-y

**Published:** 2025-09-18

**Authors:** Xavier Fablet, Thierry Dreano, Francisco Llamas Gutierrez, Warren Kim, Raphaël Guillin

**Affiliations:** 1 CHU de Rennes (Service d’imagerie Médicale), 16 Boulevard de Bulgarie, 35000 Rennes, France; 2 CHU de Rennes (Service de Chirurgie Orthopédique), 2 Rue Henri Le Guilloux, 35000 Rennes, France; 3 CHU de Rennes (Service d’anatomopathologie), 2 Rue Henri Le Guilloux, 35000 Rennes, France; 4 Centre de La Main de Bretagne, 7 Boulevard de La Boutière, 35760 Saint-Grégoire, France

**Keywords:** Ultrasounds, Distal posterior interosseous nerve, Orthopedic surgery, Neuroma, Acrel’s ganglion, Wrist pain

## Abstract

A distal posterior interosseous nerve (DPIN) neuroma related to prior surgery of the wrist can lead to disabling chronic pain. Ultrasound may represent a useful diagnostic tool due to its high resolution and ability to detect iatrogenic neuromas along small nerves of the limbs. However, the utility of ultrasound in the evaluation of both the normal sonographic appearance of the DPIN and traumatic neuromas is limited. We present a series of two patients who suffered from chronic dorsal wrist postoperative pain, where ultrasound detected histologically confirmed DPIN neuromas. After DPIN resection, the pain of our two patients completely disappeared without any functional deficit.

## Introduction

The posterior interosseous nerve has a predominant motor function dedicated to wrist and finger extension, but also plays a sensory role as its distal branch (DPIN) innervates the dorsal wrist capsule [1–9]. Due to its position, the DPIN may be injured by the surgeon during the dorsal approach of the wrist. This may result in future debilitating symptoms which, as for other small distal nerves, may be overlooked for years before being diagnosed [10].

In this regard, ultrasound has been shown to be a useful diagnostic tool in both exploring small nerves of the limbs and diagnosing the presence of iatrogenic neuromas related to the high resolution achieved with modern devices [11]. In the upper limb, Causeret has reported the superficial branch of the radial nerve and the dorsal cutaneous branch of the ulnar nerve to be both the most frequently injured by surgeons and easily accessible by the probe [12].

Currently, there are only a few reports of normal [13] or pathologic appearances of the DPIN [14, 15] assessed by ultrasound in the literature.

This work presents two cases of surgically and histologically confirmed iatrogenic neuroma of the DPIN. It also discusses the contribution of US in the diagnosis of such a condition, which may be easily overlooked clinically.

## Case 1

**Fig. 1:**
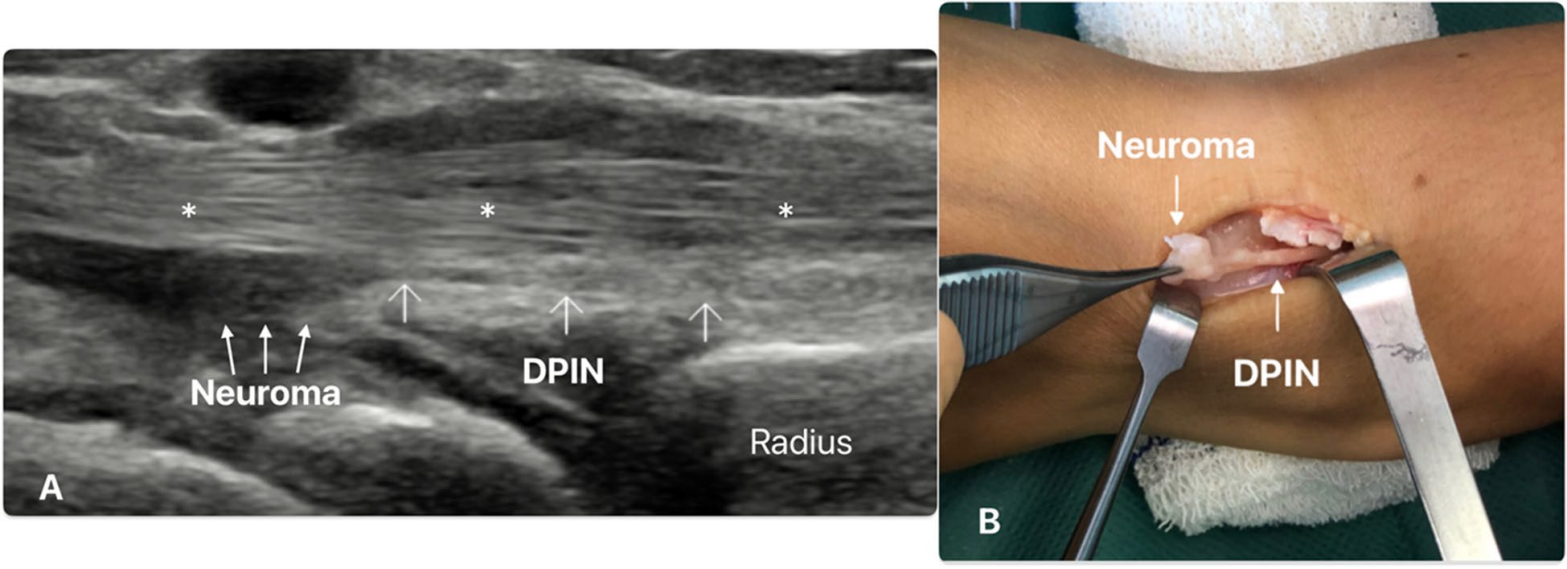
**A** Longitudinal view of the DPIN terminal neuroma (left, distal; right, proximal). Hypoechoic terminal stump neuroma of the distal posterior interosseous nerve (DPIN) beneath the extensor tendons of the fourth compartment (*) and over the first row of the carpus. **B** Operative view of the dorsal wrist (left, distal; right, proximal) showing the distal posterior interosseous nerve terminal neuroma during resection surgery

The first patient is a 29-year-old woman who works as a cashier. She underwent two resections of arthrosynovial cysts on the back of one wrist within the last 4 years. The first surgery was performed using an open approach, while the second was performed arthroscopically with capsulodesis according to Mathoulin’s technique [16]. She was referred 2 years later with persistent dorsal and central wrist pain, localized around the scar and triggered by pressure, with ascending pain and allodynia. Ultrasonographic exploration, using a high-frequency L4-18A superficial probe (RS85, Samsung Medical Co., Ltd., Seoul, South Korea), identified a stump of the DPIN, suggesting the presence of a terminal neuroma located underneath the tendons of the fourth extensor compartment and over the first row of the carpus (Fig. 1). It measured 2 mm in the short-axis. Palpation with the probe reproduced the patient’s pain and suggested an ultrasonographic-Tinel sign. For diagnostic purposes, an ultrasonographic-guided injection of a local anesthetic solution (2 ml of Xylocaine 1%) and corticosteroid (0.5 ml of Hydrocortancyl) was attempted, but without significant clinical improvement. Because the contribution of the neuroma to the patient’s pain was highly suspected, a surgical exploration was performed which identified a terminal neuroma that was confirmed by histopathological analysis after resection (Fig. 1). This surgery successfully eliminated all of the patient’s reported pain.

## Case 2

**Fig. 2:**
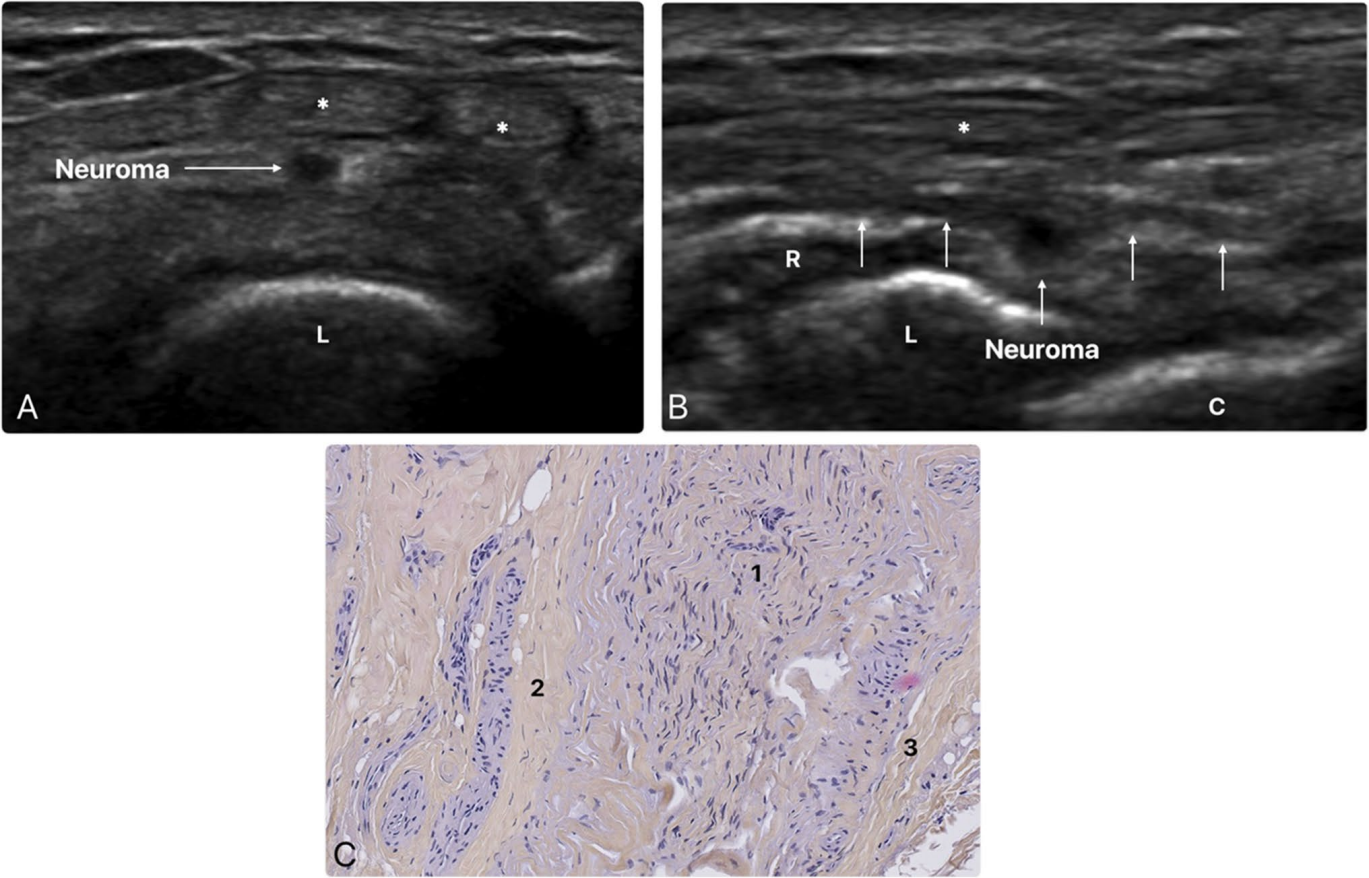
**A** and **B** Axial and sagittal ultrasonographic views of the DPIN (white arrows) with spindle post-traumatic neuroma over the lunate (*, tendons of the third and fourth extensor compartments; R, radius; L, lunate; C, capitate). **C** Histological correlation of the DPIN neuroma. Proliferation of nerve fibers (1) surrounded by a thickened endoneurium (2) and a fibrous perineurium (3) (×10 magnification)

The second patient, a 51-year-old woman, works in a laundry. She underwent resection of an accessory carpal bone, probably an os epilunate, and returned 4 years later due to the onset of new wrist pain, which appeared at least 2 years after surgery. The pain originated from the radial side of the back of the wrist, near Lister’s tubercle, and radiated toward the dorsal sides of the thumb and index finger. The pain was primarily nocturnal and partially relieved by wearing a brace. No other traumatic event was reported. Initial clinical assessment, supported by wrist MRI findings demonstrating a small amount of fluid at the interface between the extensor pollicis longus tendon and the tendons of the second dorsal compartment, not detected by ultrasound, was suggestive of distal intersection syndrome. However, an ultrasonographic-guided injection of a mixture of anesthetics and steroids between the tendons of these two compartments, using a high-frequency L18-5 superficial probe (IU22, Philips, Amsterdam, Netherlands), did not result in improvement of symptoms. The patient underwent a second ultrasonographic evaluation a few months later, using a high-frequency L18-5 superficial probe (IU22, Philips, Amsterdam, Netherlands), which this time emphasized a focal fusiform thickening of the DPIN at the level of the distal border of the lunate (Fig. 2). Thickening reached 2 mm in the short axis of the nerve and 4 mm along its long axis. This finding, which was missed during the first US due to a lack of awareness of this nerve, suggested the presence of a spindle post-traumatic neuroma. An ultrasonographic-Tinel sign was present while diagnostic ultrasonographic-guided injection of corticosteroids (half an ampoule of Cortivazol 3.75 mg/1.5 ml) and levobupivacaine (2 ml of Chirocaine 5%, AbbVie) around the DPIN provided symptomatic relief for 15 days. These findings led to a surgical excision, which showed the presence of a fusiform thickening of the DPIN adhering to the wrist capsule. This diagnosis was once again confirmed by histopathological analysis, which was consistent with post-traumatic neuroma (Fig. 2). DPIN resection resulted in the complete disappearance of symptoms.

## Discussion

Neuromas of the DPIN, unlike neuromas of the superficial branch of the radial nerve [17–21], have been infrequently reported in the literature [6, 9, 10, 22]. This lack of recognition by the medical community may explain the diagnostic delay observed in affected patients. In our subjects, this delay reached 18 and 42 months, respectively, while it was 6 months in the case report by Dellon and Seif [9], 4 months to 2 years for Carr [6], 1 year for Pérez [22], and 7 months to 15 years according to Loh [10].

The contribution of open or arthroscopic surgeries to the development of DPIN neuromas, as seen in our two patients, is similar to the findings reported in several previous studies [9, 10, 23, 24]. Regarding open procedures, both studies from Loh and Dellon and Seif reported several cases of DPIN neuromas after excision of arthrosynovial cysts or scapholunate capsulodesis [9, 10]. Regarding arthroscopic procedures, Cheah demonstrated a high likelihood of DPIN injury during 3–4 portal arthroscopic approaches, occurring in half of the cases, even when performed by an experienced surgeon [23]. Del Piñal reported a case of a complete resection of the DPIN with this same portal [24]. Additionally, our first patient underwent capsulodesis according to Mathoulin’s technique, which uses the midcarpal ulnar, midcarpal radial, and 3–4 portals that are prone to DPIN injury [25]. However, the overall occurrence of this type of injury remains uncommon, as a literature review identified only one complete DPIN accidental transection out of 899 arthroscopies [26], while a retrospective multicenter study of over 10,000 arthroscopies reported only three injuries without specifying the involved approaches [27].

Beyond surgical contexts, other mechanisms of injury have been reported, including trauma with distal forearm fractures, microtrauma from repeated wrist dorsiflexion, or compression secondary to synovial involvement in rheumatoid arthritis [6, 22, 28–30].

The clinical manifestation of DPIN injury differed between our two patients. The first patient primarily experienced central wrist pain with an ascending pattern, while the second patient described radial pain radiating distally to the thumb and index finger. The first patient’s clinical presentation is similar to that previously reported in the literature [6, 9, 10]. For Carr and Dellon and Seif, pain was located on the dorsal and distal side of the wrist, mostly ulnar to Lister’s tubercle and without clear radiation. However, Loh also encountered cases with radiating pain towards the dorsal sides of the thumb, index, and middle fingers. Involvement of such a radial territory may be explained by anatomy, as anastomoses frequently occur between the PIN and superficial branches of the radial nerve [31].

The cases presented in this study demonstrate the usefulness of US in the diagnosis of DPIN neuromas, as has been observed for many other upper and lower limb peripheral nerves [12]. Specifically, ultrasonography is consistently able to depict this nerve at the wrist, as demonstrated by Smith in a series of twenty cases [13]. Furthermore, this tool is able to depict both spindle and terminal type neuromas of small peripheral nerves as a hypoechoic thickening related to axonotmesis or neurotmesis injuries, respectively [11, 32, 33]. In the literature, only two cases of traumatic DPIN injuries identified via US are available. The first is described as a hypoechoic neuromatous thickening of the DPIN following a wrist trauma without further details, and the second is characterized by hypoechoic scar tissue surrounding the DPIN after the excision of an arthrosynovial cyst [14, 15]. Unlike the two patients reported in the present study, the final diagnosis was not confirmed histologically, and such a diagnosis may be confused with an anatomical thickening of the nerve named the “Acrel’s ganglion” [34].

Acrel’s ganglion, which represents a main pitfall for the diagnosis of iatrogenic neuroma of the DPIN at the wrist, may be present in many normal subjects, as Tubbs has encountered it in all of their studied wrists [34]. It can be located from Lister’s tubercle proximally to the level of the articular capsule distally, shares the same histological composition with a peripheral nerve, without any signs of irritation or chronic inflammation, and its origin remains undetermined [1, 6, 8, 24, 34–39]. To date, no ultrasound studies have specifically investigated this anatomical thickening.

However, when a swelling of the nerve is identified with US, several signs may suggest its neuromatous origin rather than an Acrel’s ganglion. First, the presence of a “ultrasound Tinel sign” that results in typical triggering of pain upon the sonographic probe [15, 40]. This feature was observed in both patients in the present study. A positive response to an anesthetic or steroid infiltrative test may also offer a clue for the diagnosis [41–43]. However, this is only observed in one of our two cases, which highlights the relative subjectivity of the procedure. Finally, the presence of a hypoechoic scar encasement may also suggest the traumatic origin of a thickening of the nerve despite the low prevalence of this feature [11].

After DPIN resection, painful symptoms completely resolved in both of our patients without any motor or skin sensory deficits. This is consistent with the literature as innervation of the skin at the wrist results from the cutaneous branches of the radial and ulnar nerves [1, 35, 39].

In conclusion, this case report demonstrates the ability of ultrasound to identify iatrogenic neuromas of the distal posterior interosseous nerve at the wrist in order to explain postoperative pain after dorsal wrist surgery. Despite the low value of each sign documented in our two patients, a history of trauma or wrist pain, a positive Tinel sign on ultrasound, and symptom improvement following a targeted steroid injection may help to discriminate this iatrogenic lesion from an Acrel’s ganglion.

## Data Availability

Image collection: Ultrasound images were extracted from the clinical PACS (Telemis, Louvain-la-Neuve, France) at the CHU of Rennes using Digital Imaging and Communications in Medicine (DICOM) query/retrieve software. All image data were de-identified and stored in DICOM standard format. Per operative view image: This photo was taken by the orthopedic surgeon during distal posterior interosseous nerve resection. Histological image: The histological image in the second figure has been provided by the pathologist from his personal analysis of the specimen.
